# Acute Upper Extremity Ischemia Due to Cardioembolism From Undiagnosed Atrial Fibrillation

**DOI:** 10.7759/cureus.21148

**Published:** 2022-01-12

**Authors:** Shin T Zaw, Thinzar Zaw, Edwin C Pigman

**Affiliations:** 1 Medical School, Lake Erie College of Osteopathic Medicine, Bradenton, USA; 2 Medical School, University of Central Florida College of Medicine, Orlando, USA; 3 Emergency Medicine, AdventHealth Sebring, Sebring, USA

**Keywords:** pad, thrombectomy, acute limb ischemia, embolism, atrial fibrillation

## Abstract

The current study presents a case of right upper extremity ischemia secondary to cardioembolism in an elderly female with active and previously undiagnosed atrial fibrillation. The patient had no past medical history of any chronic cardiac disease or significant cardiac events. Computed tomographic angiography (CTA) was not performed due to her allergy to contrast material. A non-contrast computed tomography (CT) revealed mild atherosclerotic calcification of the right brachiocephalic artery; however, dissection or mural thrombus of the inflow vessels could not be ruled out. In evaluating a patient with acute ischemia of the upper limb, it is essential to obtain a complete history, including allergies, and be prepared to use alternative techniques for assessing arteries, if necessary. Routine cardiac function testing should also be prioritized in all elderly individuals, even those with no previous history of cardiovascular disease or symptoms.

## Introduction

Peripheral arterial events can lead to high case fatality risk and moderate or severe functional disability if emergency vascular or surgical intervention is not performed. This study presents a case of acute right upper extremity pain secondary to occlusion at the level of brachial artery bifurcation in an elderly female, which occurred in the setting of atrial fibrillation. Moreover, cardiac events are more likely to cause upper extremity ischemia due to the formation of mural thrombi [1]. The presence of poikilothermia, paresis, pulselessness, and pallor was further investigated by a non-contrast CT and arteriovenous duplex ultrasound. As the patient was allergic to contrast material, CTA was not performed. However, many alternative methods with reasonable specificity and sensitivity are available for such patients, including magnetic resonance angiogram (MRA) without contrast and duplex ultrasonography.

## Case presentation

The patient is a 74-year-old female who presented to an outside rural hospital emergency department after experiencing acute, severe atraumatic pain in the right upper extremity that started while writing a cheque. She reported severe pain originating in the antecubital region of her right extremity, radiating down to her right hand. The patient described the pain as a throbbing sensation and rated it an eight on a pain scale of one to 10. It had no aggravating or relieving factors. Her medical history included insulin-controlled type 1 diabetes, hypertension, hypothyroidism, and hyperlipidemia. Additionally, the patient did not have any previous episodes of claudication, peripheral arterial disease, coronary arterial disease, congestive heart failure, or cardiac dysrhythmia. She is a non-smoker who is allergic to intravenous contrast. 

**Figure 1:**
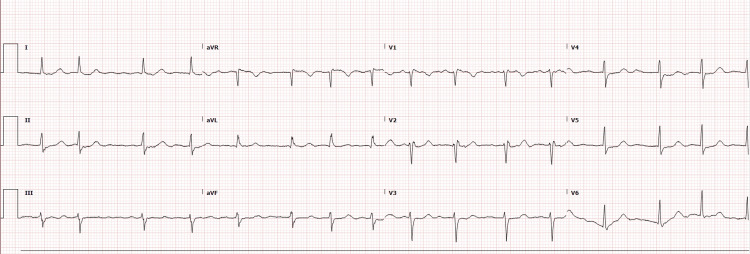
ECG demonstrating atrial fibrillation

**Figure 2:**
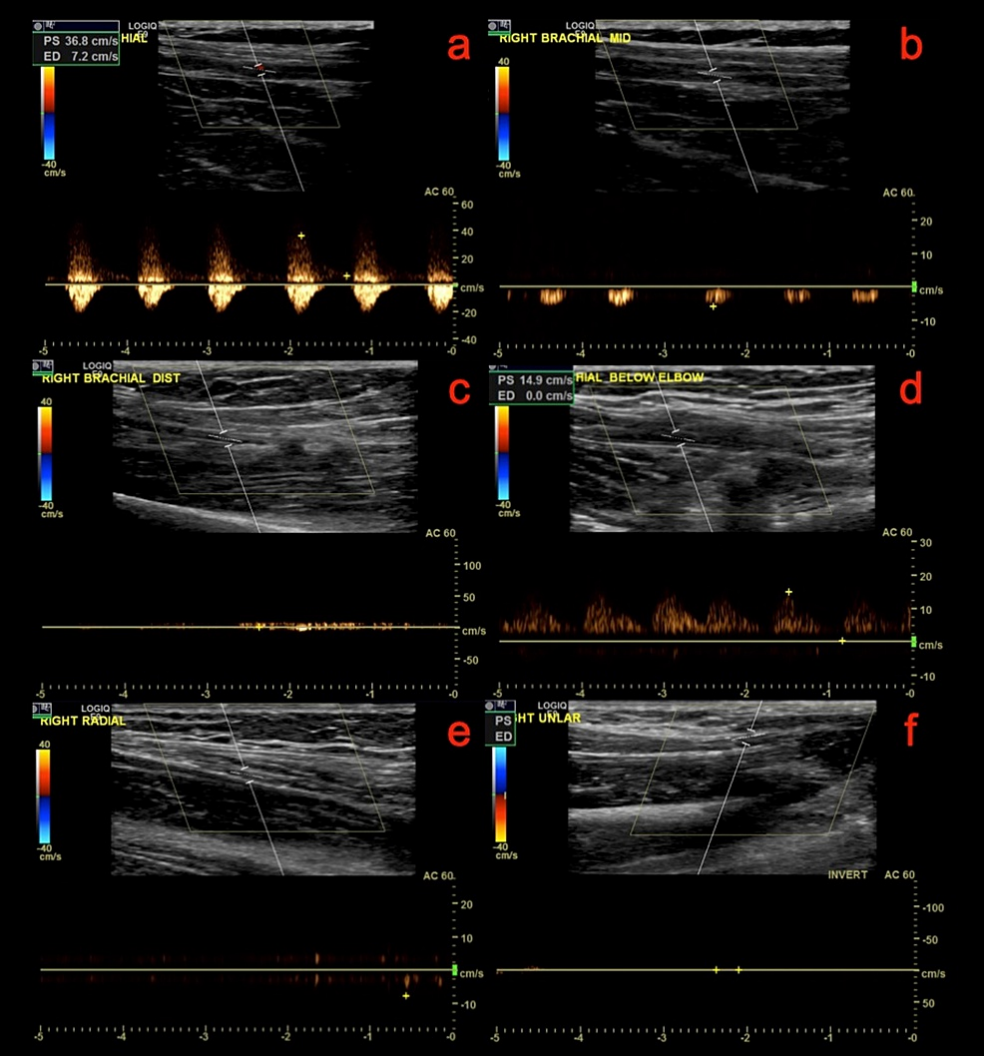
Arterial Duplex Ultrasound evaluation at different locations: a) right brachial proximal; b) right brachial mid; c) right brachial distal; d) right brachial distal below the elbow; e) right radial; f) right ulnar

On examination, her right hand was cold, blue, and numb compared to the left. There was a delay in the capillary refill. The brachial and radial pulses in her right upper extremity were barely palpable, but they were strong in her left upper extremity. Her pulse rate was 93 beats/min. Laboratory investigations revealed a blood glucose level of 246 mg/dl, a white blood cell count of 12 K/uL, and a hemoglobin concentration of 12.3 g/dL. It was suspected that she had limb ischemia secondary to cardioembolism. An electrocardiogram (ECG) obtained revealed atrial fibrillation with a ventricular response rate of 93 beats/min (Figure 1). Arteriovenous duplex ultrasound of the right upper extremity was also obtained. However, venous duplex ultrasound was unremarkable. Arterial duplex ultrasound demonstrated 36.8/monophasic in the proximal portion, and there was normal arterial flow in the mid brachial artery. Peak systolic velocity decreased to 15 cm/s/monophasic in a small segment of the distal brachial artery below the elbow. No flow was present in the radial and ulnar arteries (Figure 2).

Following the initial ultrasound and ECG, vascular surgery was consulted, and a CTA was requested. As the patient was allergic to contrast dye, CTA was not performed. However, a non-contrast CT was ordered instead, which indicated mild atherosclerotic calcification of the right brachiocephalic artery. The results were mildly inconclusive due to the inability to rule out a possible dissection or mural thrombus of the inflow vessels. However, a bolus of heparin 5,000 units was administered, and an intravenous drip of heparin 25,000 units was initiated immediately afterward. Due to her volume depletion, she was given an IV analgesic and hydration and transferred to a different facility. Cardiology was consulted, and an echocardiogram was ordered, which also revealed atrial fibrillation but was otherwise unremarkable. The surgery was performed as an emergent open thrombectomy of the right brachial artery, and it went off without complications. The patient was started on Eliquis 5mg twice a day (BID) and Cardizem 240mg every day (QD) postoperatively. Also, the patient was followed up by her physician and has remained stable. Tests were ordered to screen for hypercoagulable states, and results indicated unremarkable prothrombin time/international normalized ratio (PT/INR), partial thromboplastin time (PTT), D-Dimer, Angiotensin II (ATIII), protein C, protein S, Factor V Leiden, lupus anticoagulant, prothrombin gene mutation, Janus Kinase 2 (JAK-2) mutation, Myeloproliferative leukemia (MPL) mutation, AntiCardiolipin antibodies, and anti-beta-2-glycoprotein antibodies. 

## Discussion

Most cases of limb ischemia occur in the lower extremities. However, this case was unique and rare as ischemia was present in the upper extremity. Since the most likely source of upper extremity ischemia is a cardiac source, an ECG was performed first to determine the root cause of the embolus. Atrial fibrillation was diagnosed and was determined to be the root cause of embolus, as static blood is more likely to throw emboli. Due to the patient's allergy to contrast material, a CTA was avoided in favor of duplex ultrasonography. This procedure evaluates the level of arterial occlusion, which was the bifurcation of the brachial artery for this patient. A CTA is the most frequently used imaging modality in hospitals since its imaging quality is comparable to arteriograms. However, in cases where CTA cannot be used, a duplex ultrasound provides an effective alternative. A low-profile, 10-MHz linear array transducer was used to perform the duplex ultrasonography [2]. The patient presented with significant pain in addition to the pallor of the right upper extremity, paresthesia, and poikilothermia, as a healthy artery without established collaterals, was blocked. A complete physical and vascular examination of the extremity was performed to classify the ischemia and determine the best treatment strategy.

Embolism is defined as debris in the vascular system obstructing a distal artery. Lower extremity ischemia accounts for the majority of limb ischemia cases. Only 2-18% of limb ischemia episodes are of the upper extremity [3]. The most common cause of acute upper extremity ischemia is emboli from a cardiac source. A mural thrombus mostly obstructs peripheral distal arteries, causing acute disruption of blood flow [4]. Moreover, the most common site of obstruction is at the origin of the profunda brachialis artery or the bifurcation of the brachial artery. Other causes include thrombosis, atherosclerotic stenosis, arterial trauma, arterial dissection, and acute thrombosis of a stent or graft. Proximal atherosclerotic debris is a source of embolus-debris from the proximal aorta that breaks and lodges in the peripheral vessels [5]. Rare presentations of upper extremity ischemia include dialysis access steal syndrome and ischemic monomelic neuropathy. However, axillary-femoral stump syndrome is another rare etiology of upper extremity ischemia [6]. 

The recent coronavirus disease 2019 (COVID-19) outbreak caused lupus anti-coagulant syndrome, and rare cases of limb ischemia have been reported in COVID-19 patients [7]. Moreover, open injury to the brachial artery can also result in an ischemic hand. In closed injuries, hypothenar hammer syndrome occurs when there is trauma to the ulnar side of the palm [8]. When embolism occurs, it first manifests as a sensory deficit, followed by a motor deficit and muscle weakness. Also, when there is an acute embolism, the limb becomes white, and a neurosensory deficiency occurs because there are no established collaterals in a healthy artery [9]. Rutherford classifies acute limb ischemia into three types based on clinical findings and Doppler measurements. These are viable extremities, threatened extremities, and irreversible defects. When irreversible defects occur, amputation is considered instead of revascularization [10]. 

Duplex ultrasonography is a very valuable diagnostic procedure. In one study, the sensitivity and specificity of Duplex ultrasonography (US) for determining renal artery stenosis (RAS) were found to be 80/54% on a patient basis; however, for the same purpose, it was 100/56% for CTA. Thus, MRA and CTA are found to be significantly better than duplex US [2].

## Conclusions

When evaluating a patient with acute ischemia of the upper limb, it is essential to thoroughly assess the patient by taking a complete history, including any previous allergic reactions to drugs or contrast materials, and performing rapid diagnostic tests to preserve the patient’s limb and life. If the patient is allergic to CTA contrast, alternative techniques such as ultrasonography and non-contrast CT are performed to evaluate the arteries. These methods have low precision compared to CTA, but they are still the gold standard tests for such situations. This case also emphasizes the importance of routine cardiac function testing in the elderly, despite no previous history of cardiovascular disease or symptoms consistent with arrhythmia.
